# Supplementary figures and images for: The Efficiency of Selected Green Solvents and Parameters for Polyphenol Extraction from Chokeberry (Aronia melanocarpa (Michx)) Pomace
Source: Foods. 2023 Oct 1;12(19):3639. doi: 10.3390/foods12193639 (PMC10572178; doi:10.3390/foods12193639)

(a)

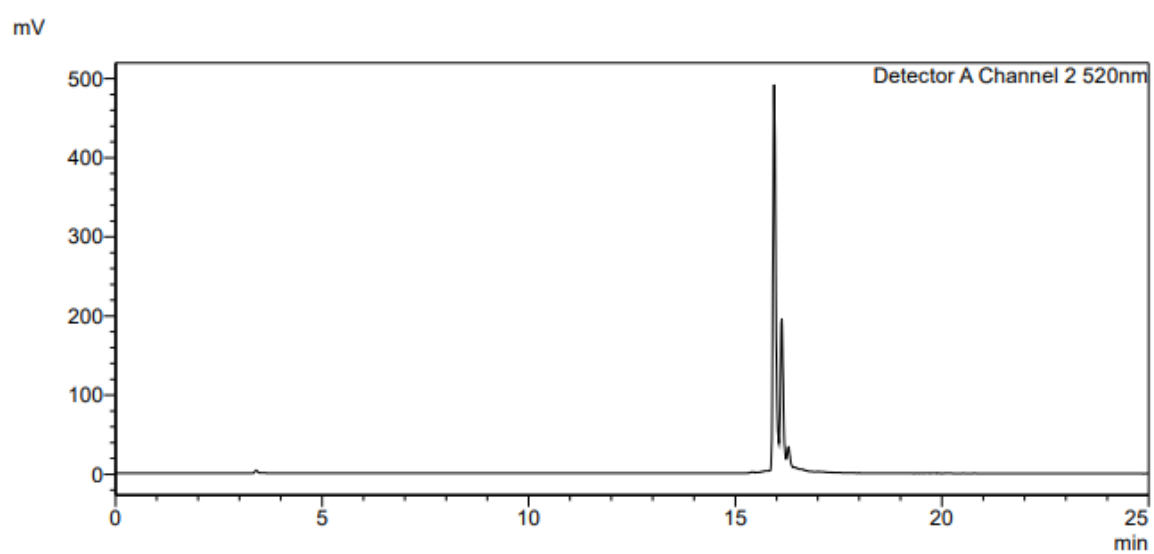

(b)

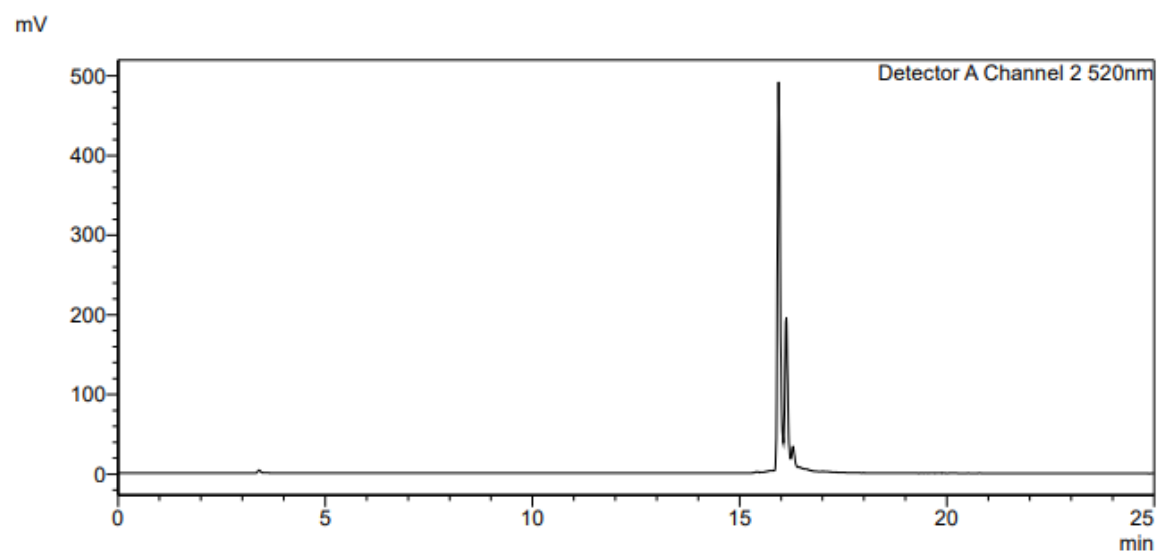

(c)

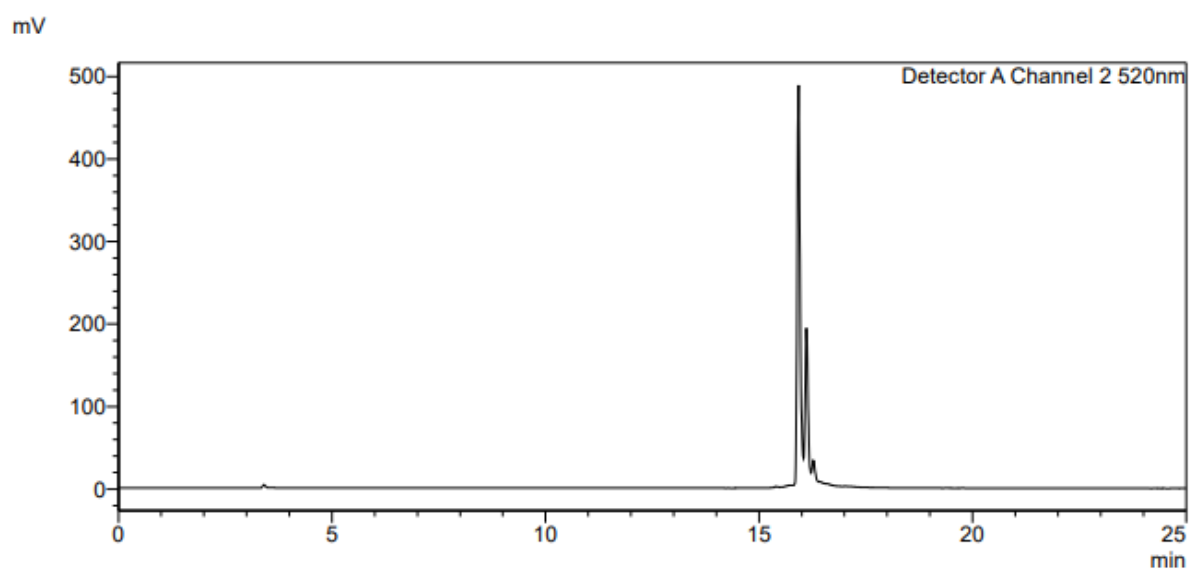

(d)

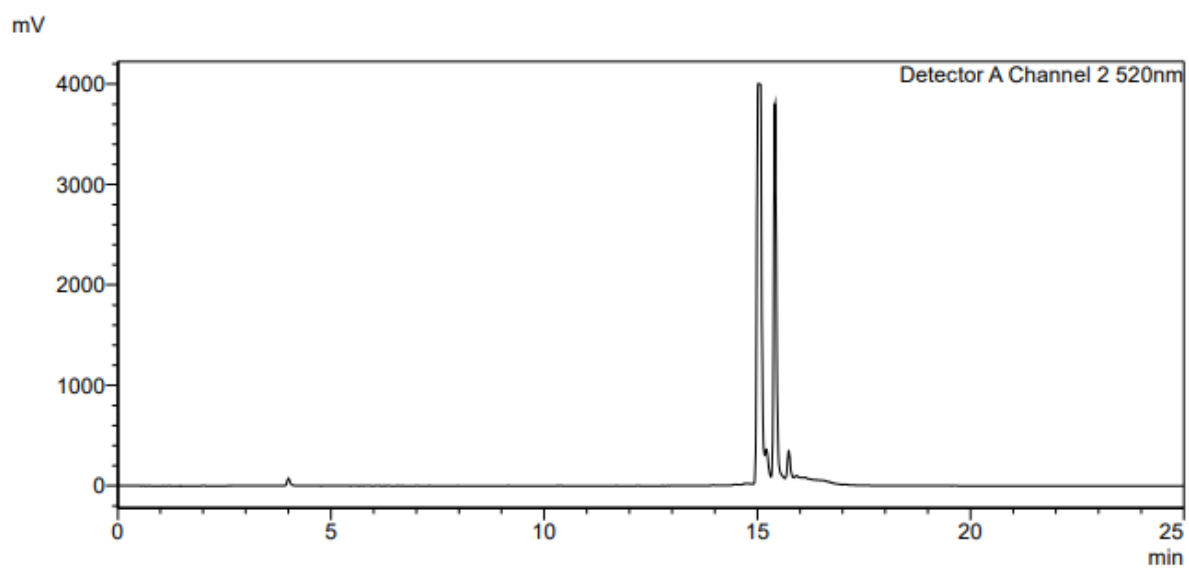

(e)

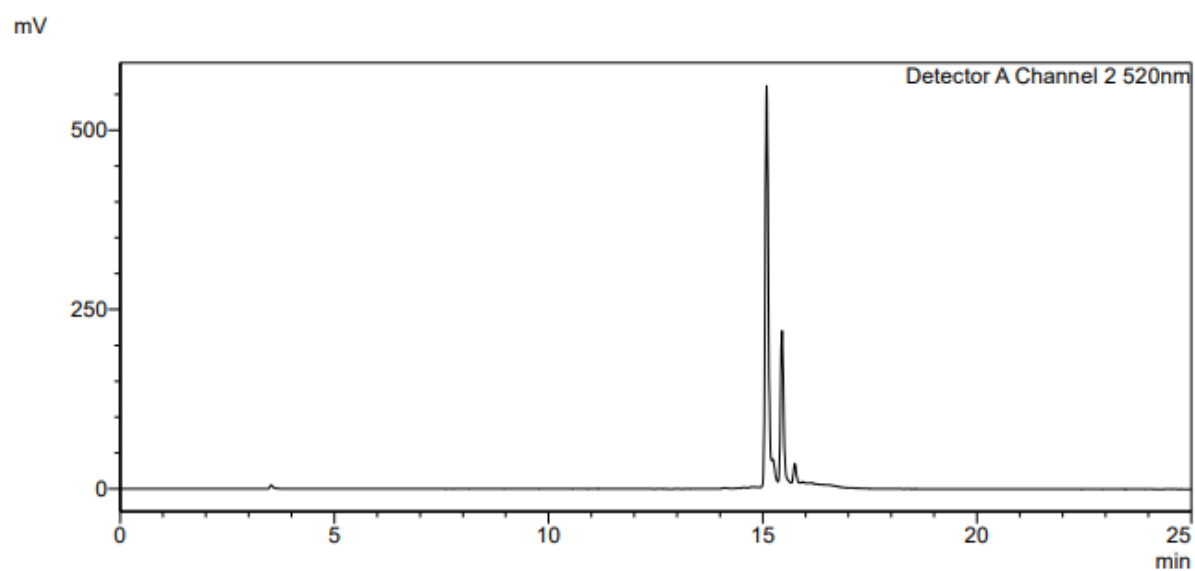

(f)

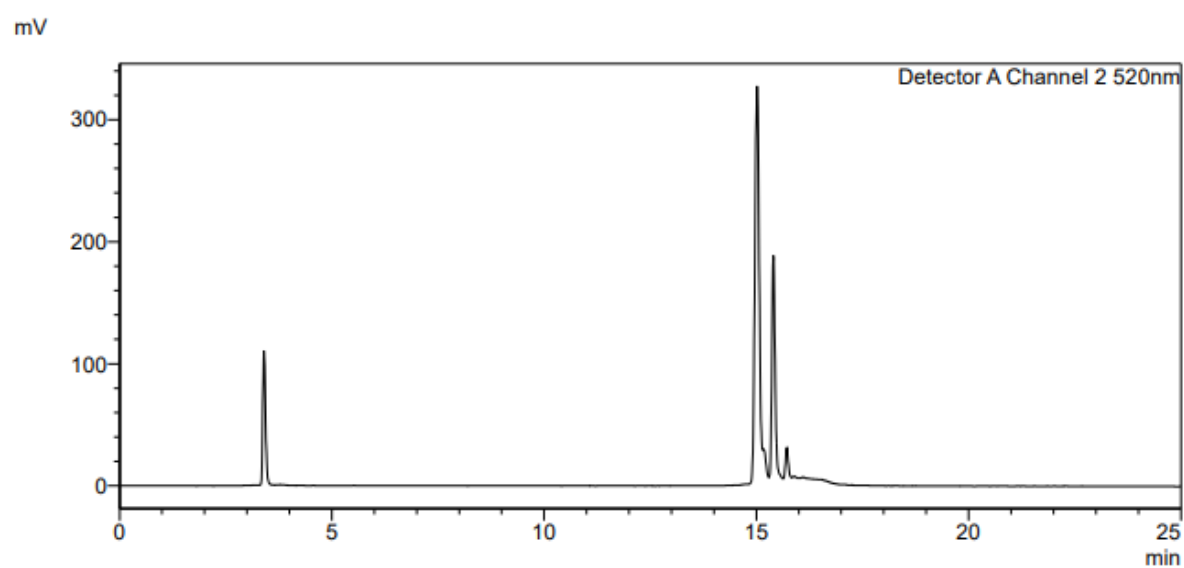

(g)

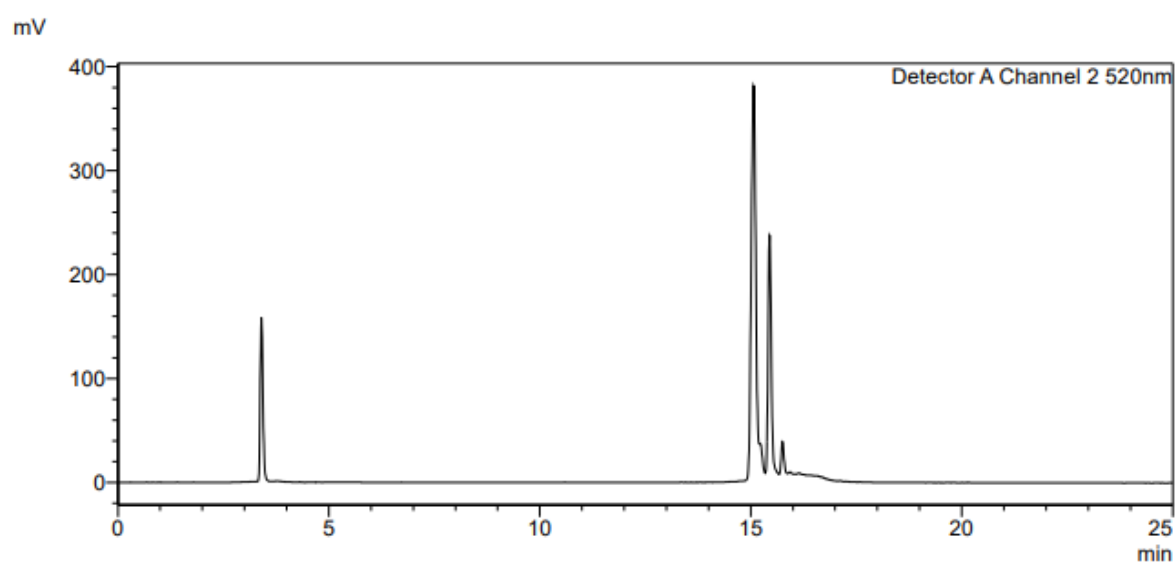

(h)

mV

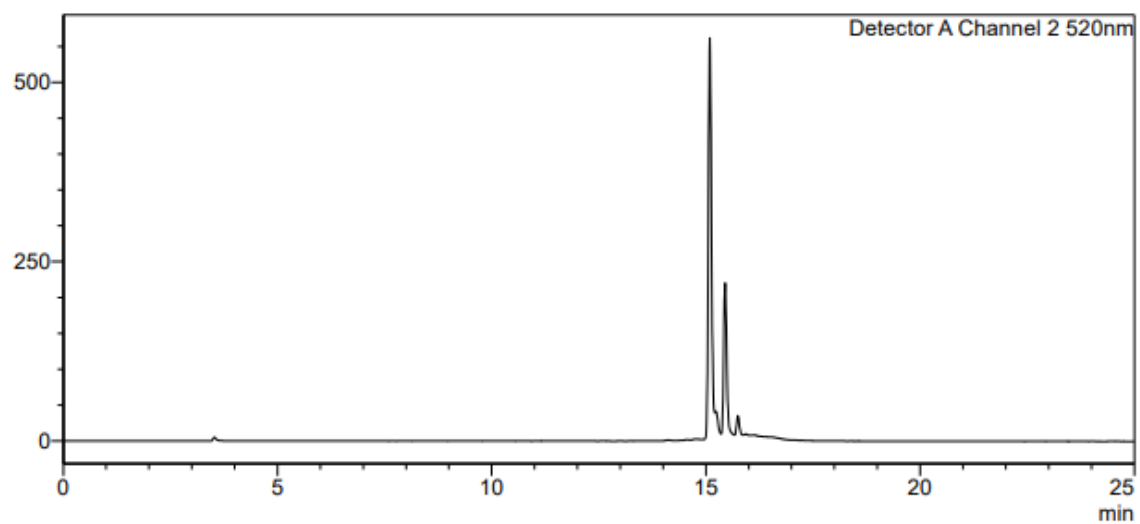

(i)

mV

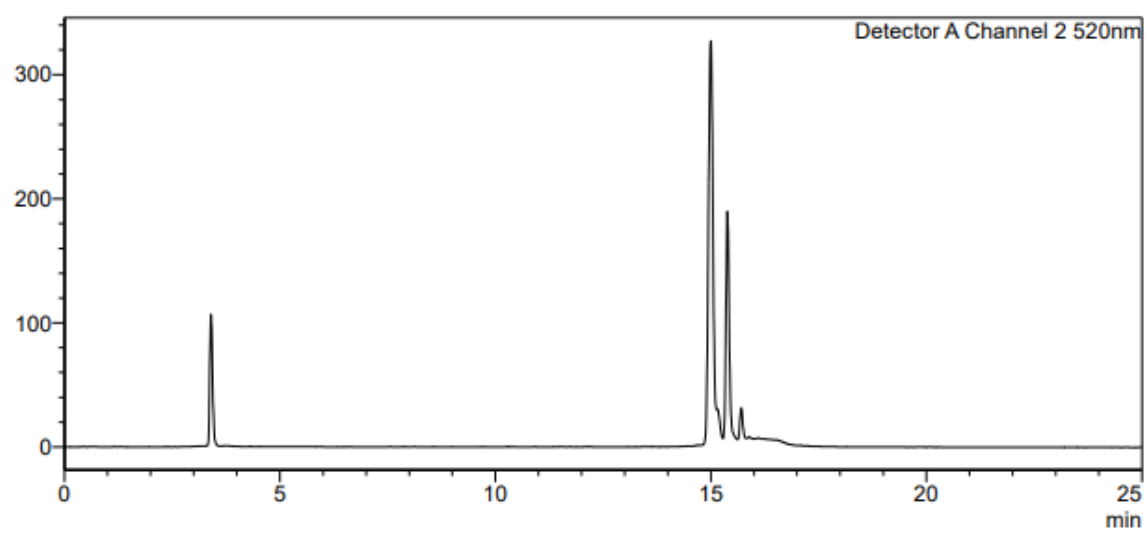

(j)

mV

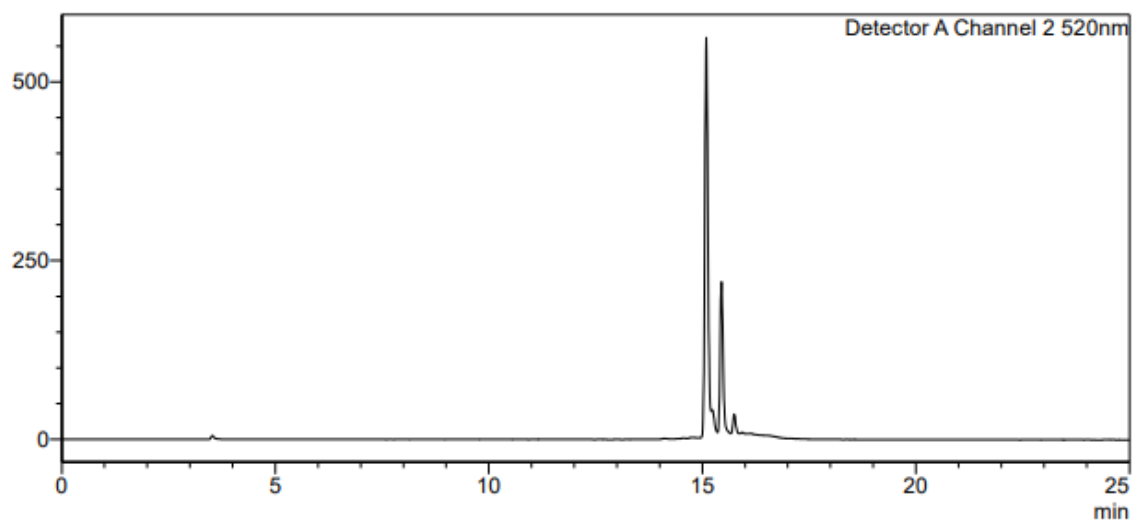

Figure S1. Anthocyanin chromatograms of different chokeberry extracts

Supplement: Supplementary file 1 [file foods-12-03639-s001.zip › foods-2626387-supplementary.pdf]
